# Mortality and bleeding associated with the management of sub-massive pulmonary embolism: a systematic review and Bayesian network meta-analysis

**DOI:** 10.1038/s41598-023-34348-9

**Published:** 2023-05-03

**Authors:** Don Mathew, Jay Kim, Bhanu Prasad Kosuru, Deepthi Devagudi, Akil Sherif, Utsav Shrestha, Prabhjot Bedi

**Affiliations:** 1 Department of Internal Medicine, University of Pittsburgh Medical Center (UPMC), Pittsburgh, PA USA; 2 Department of Internal Medicine, West Anaheim Medical Center, Anaheim, CA USA; 3 Department of Cardiology, St Vincent Hospital, Worcester, MA USA; 4 Department of Pulmonary and Critical Care Medicine, West Virginia University, Morgantown, WV USA; 5 Department of Internal Medicine, UPMC East, 2775 Mosside Blvd, Monroeville, PA 15146 USA

**Keywords:** Cardiology, Outcomes research

## Abstract

Current guidelines recommend anticoagulation (AC) for low and intermediate-risk pulmonary embolism (PE) and systemic thrombolysis (tPA) for high risk (massive) PE. How these treatment options compare with other modalities of treatment such as catheter directed thrombolysis (CDT), ultrasound assisted catheter thrombolysis (USAT), and administering lower dose of thrombolytics (LDT) is unclear. There is no study that has compared all these treatment options. We conducted a systematic review and Bayesian network meta-analysis of randomized controlled trials in patients with submassive (intermediate risk) PE. Fourteen randomized controlled trials were included, comprising 2132 patients. On Bayesian network meta-analysis, a significant decrease in mortality was noted in tPA versus AC. There was no significant difference between USAT versus CDT. For risk of major bleeding, there was no significant difference in relative risk of major bleeding between tPA versus AC and USAT versus CDT. tPA was found to have a significantly higher risk of minor bleeding and a lower risk of recurrent PE compared to AC. Systemic thrombolysis is associated with a significant reduction in mortality and recurrent PE compared to anticoagulation but an increased risk of minor bleeding. There was no difference in risk of major bleeding. Our study also shows that while the newer modalities of treatment for pulmonary embolism are promising, there is lack of data to comment on the purported advantages.

## Introduction

Venous thromboembolism (VTE) is the third most frequent acute cardiovascular syndrome globally behind myocardial infarction and stroke. The annual incidence of pulmonary embolism (PE) is between 39 and 115 per 100,000 population^1–4^. The current guidelines recommend anticoagulation for low and intermediate risk PE and systemic thrombolysis for high risk (massive) PE^1^. The PEITHO trial that studied systemic thrombolysis(tPA) versus anticoagulation in patients with submassive (intermediate risk) PE, found that systemic thrombolysis with Tenecteplase prevented death or hemodynamic deterioration but at an increased risk of bleeding^5^. This spurred an interest in catheter directed thrombolysis where lower doses of thrombolytics can be administered thus theoretically reaping the benefits of thrombolysis while mitigating the bleeding risks. There are two methods for catheter directed thrombolysis, standard catheter directed thrombolysis (CDT) and ultrasound assisted catheter thrombolysis (USAT). The standard catheter directed thrombolysis delivers thrombolytic agents locally through a multiple side hole catheter directly into the pulmonary artery thrombus. Ultrasound assisted catheter device (USAT) utilizes an ultrasonic core to deliver acoustic energy that helps in disrupting the thrombus thereby improving penetration of thrombolytics into the thrombus. This technique was purported to achieve similar goals of CDTs (catheter directed thrombolysis) with lower bleeding risks^6–10^. However, the purported advantages of USAT over CDT have not been demonstrated in clinical practice^11^. Recently, a few studies have suggested that administering lower dose of thrombolytics systemically (LDT) is effective while preventing bleeding complications. This modality is potentially more cost effective than catheter directed methods as it does not involve using complex equipment or the Cath lab/IR suite and saves operator involvement and time.

As there is no study to date that has compared the above treatment modalities, clinicians are relying mainly on personal preferences and institutional protocols. We hope to bridge this knowledge gap by conducting a systematic review and Bayesian network meta-analysis for treatment of submassive pulmonary embolism. The treatment modalities compared are anticoagulation (AC), systemic thrombolysis with full dose thrombolytics (tPA), low dose thrombolytics (LDT), catheter directed thrombolysis (CDT), and ultrasound assisted catheter thrombolysis (USAT). The primary outcomes studied were in-hospital mortality risk and risk of major bleeding. The secondary outcomes were risk of minor bleeding and recurrent PE. The advantages of using this method are manifold: it allows for indirect comparison of two or more treatments that have never been directly compared provided they are linked via a common comparator; it allows greater statistical precision through incorporation of indirect evidence that is not taken into account with pairwise meta-analysis; it can be used to rank treatment modalities with respect to clinical efficacy/harm, which will be useful when determining policies, guidelines surrounding the choice of treatment^12^.

## Results

**Figure 1:**
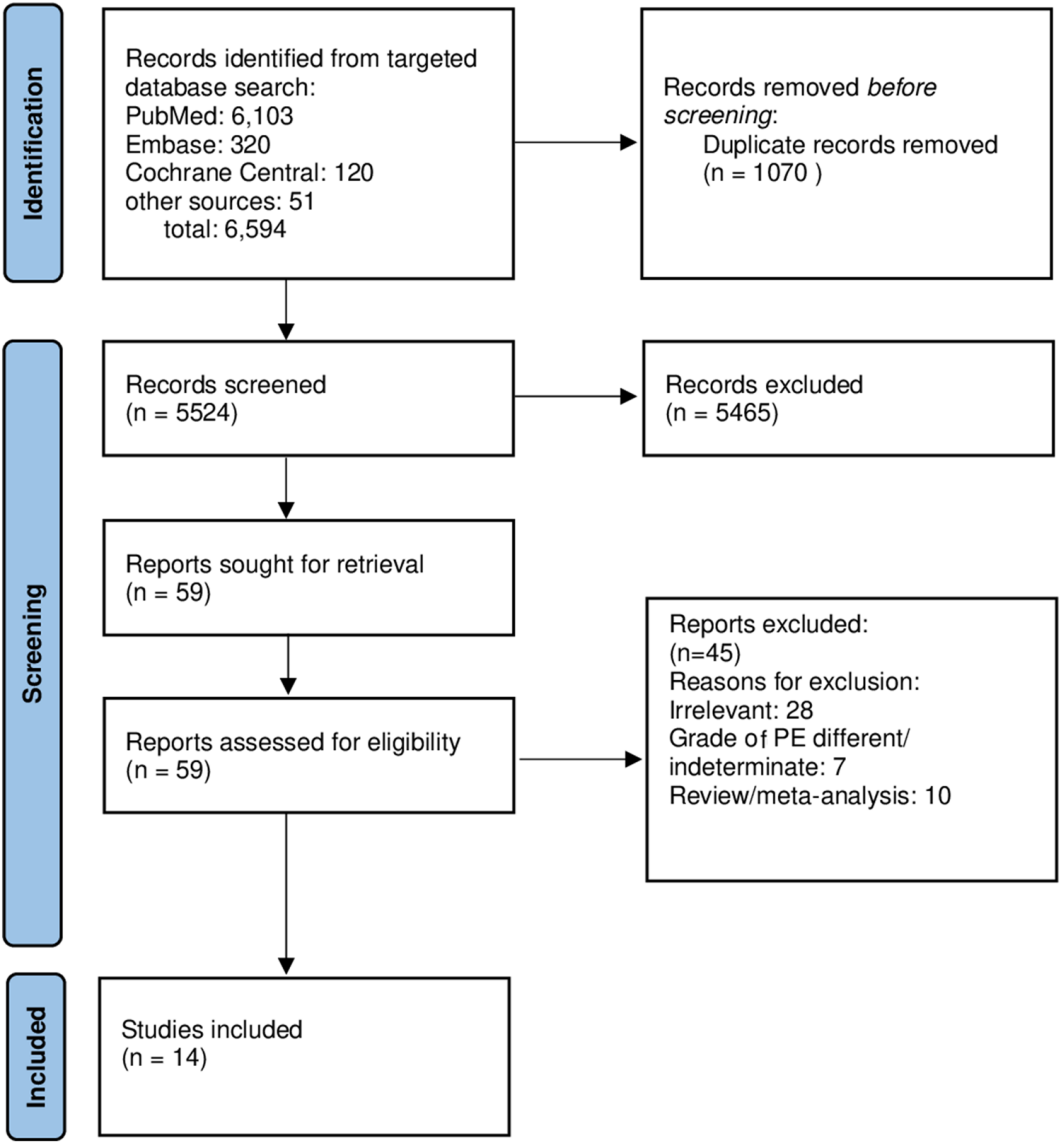
PRISMA Flow diagram.

Fourteen studies met inclusion criteria and were included in the meta-analysis^5,8,11,13–23^. Flow diagram is given in Fig. 1. Study characteristics given in Table 1.

**Table 1:** Summary of included studies.

First author, Year	Enrollment time	Sample size (T/C)	Males (T/C)	Mean Age (T/C, year)	Treatment Regimens (T vs. C)	Reported Outcomes
Levine, 1990	N.A	33/25	18/11	61.5/59.6	Alteplase versus Heparin	Mortality; major bleeding; minor bleeding; recurrent PE
Dalla-Volta, 1992	October 1988 to November 1990	20/16	7/5	65.7/63.4	Alteplase Vs Heparin	Mortality; major bleeding; minor bleeding; recurrent PE
Goldhaber, 1993	November 1988 to July 1991	46/55	16/28	58/59	Rt-PA versus Heparin	Mortality; major bleeding; minor bleeding; recurrent PE
Konstantinides 2002	September 1997 to August 2001	118/138	54/68	62.9/61.4	Alteplase versus Heparin	Mortality; Major Bleeding; minor bleeding; recurrent PE
Becattini 2010	July 2006 to December 2006	28/30	13/10	72.1/64.5	Tenecteplase versus Heparin	Mortality; Major Bleeding; minor bleeding; Recurrent PE;
Fasullo 2011	January 2005 to June 2009	37/35	21/20	55/57	Alteplase versus Heparin	Mortality; Major Bleeding; minor bleeding; Recurrent PE
Meyer 2014	November 2007 to July 2012	505/499	241/231	66.5/65.8	Tenecteplase versus Heparin	Mortality; Major Bleeding; minor bleeding; Recurrent PE;
Kline 2014	June 2008 to October 2012	40/43	20/29	57/54	Tenecteplase versus Enoxaparin	Mortality; Major Bleeding; minor bleeding; recurrent PE
Taherkhani 2014	April 2011 to November 2013	25/25	10/10	54.8/56.6	Alteplase/Streptokinase versus Enoxaparin	Mortality; Major Bleeding; minor bleeding
Sharifi 2013	May 2008 to March 2010	61/60	28/27	58/59	Low dose rt-PA versus Heparin	Mortality; Major Bleeding; Recurrent PE
Kucher 2014	November 2010 to January 2013	30/29	11/17	64/62	Ultrasound Assisted Catheter Directed Thrombolysis versus Heparin	Mortality; Major bleeding; minor bleeding; recurrent PE
Sinha, 2017	January 2012 to July 2015	45/41	14/29	54.4/55.1	Tenecteplase versus Heparin	Mortality; Major bleeding; minor bleeding; recurrent PE
Zhang, 2018	June 2014 to June 2017	33/33	18/14	60.5/58.6	Alteplase versus Heparin	Mortality; major bleeding; minor bleeding; recurrent PE
Avgerinos 2021	June 2016 to January 2020	40/41	23/20	52/55	Ultrasound Assisted Catheter Directed Thrombolysis versus Standard Catheter Directed Thrombolysis	Mortality; major bleeding; minor bleeding; recurrent PE

### Primary outcomes

The primary outcomes in our study were in-hospital mortality risk and risk of major bleeding.

**Figure 2:**
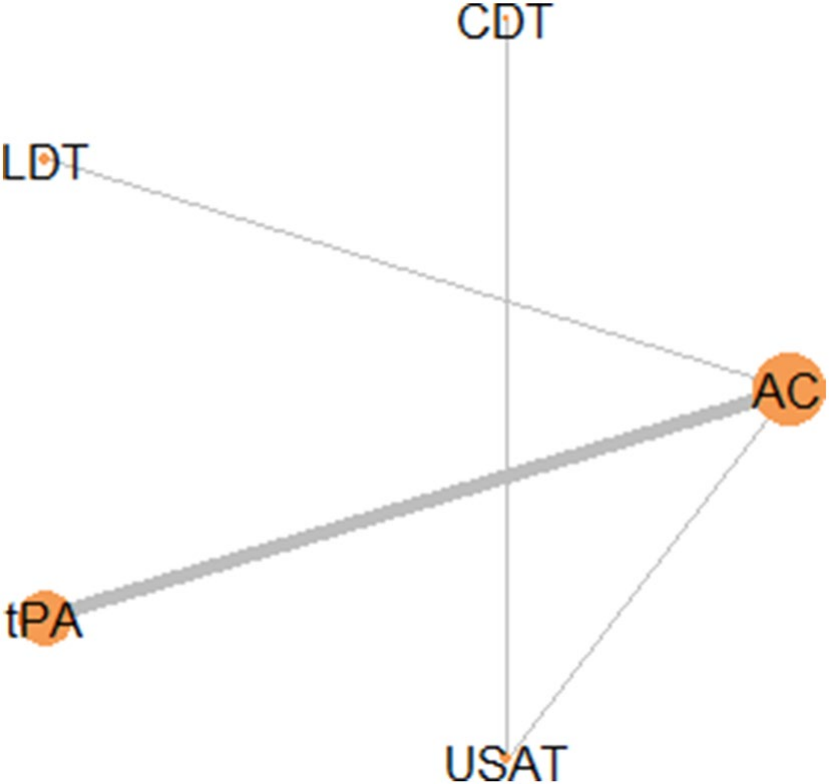
Network Plot of Studies.

In assessment of mortality risk and risk of major bleeding, fourteen studies were included comprising 2132 patients. Network plot is given in Fig. 2. Node size was based on sample size and edge width by number of studies. There were 13 studies with AC as a comparator group, 10 studies with tPA, 2 studies with USAT and LDT and one study with CDT.

### Mortality risk

**Figure 3:**
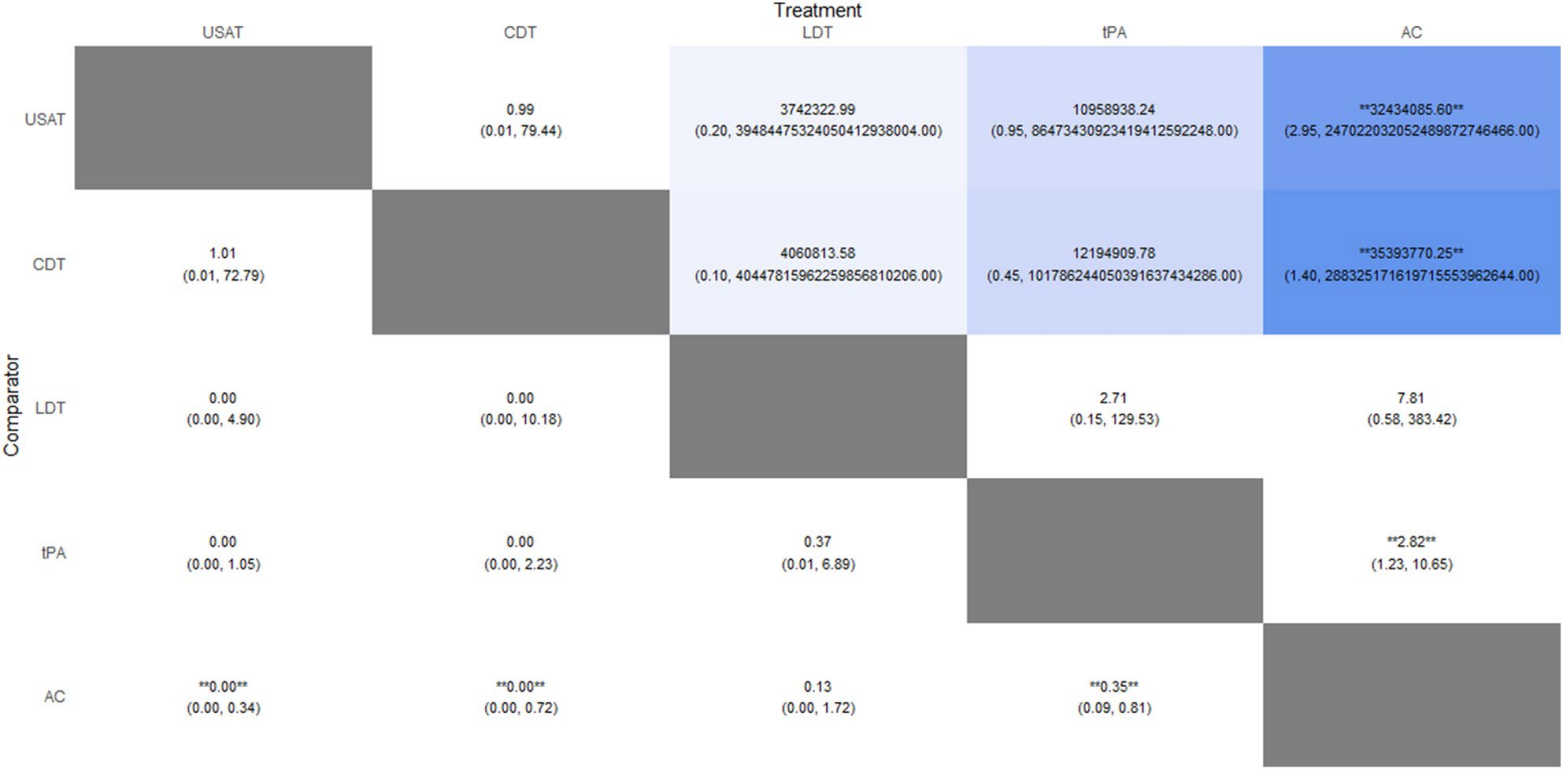
Heat Plot of In-hospital Mortality Risk.

**Figure 4:**
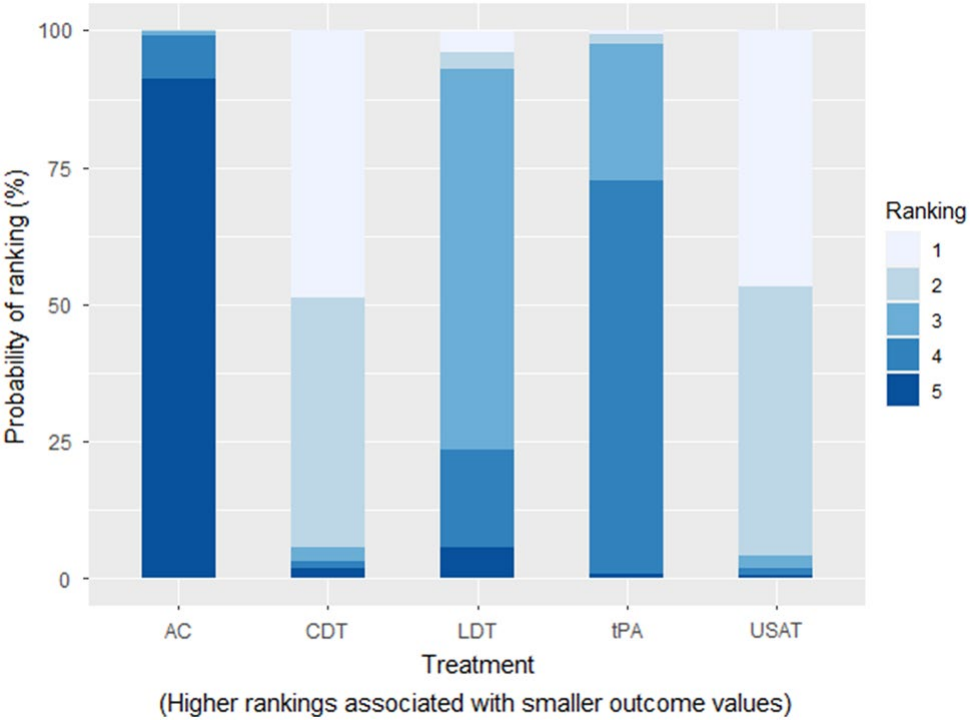
Mortality Risk Rankogram.

Compared with AC, only tPA was found to have significantly lower risk of mortality (RR: 0.35; 95% CrI: 0.09–0.81). When compared to AC, we could not draw any inference with LDT, USAT and CDT as their 95% credible intervals included zero. There was no significant difference between USAT versus CDT (RR: 1.01; 95% CrI: 0.01–72.8). No inferences could be drawn between any other comparisons as their 95% credible intervals included either zero as the lower limit or an exceedingly large number as the upper limit due to having zero events in one of the treatment arms. The Heat Plot is given in Fig. 3. On meta-regression, publication year was not found to have a significant influence (p = 0.35). The SUCRA (surface under the cumulative ranking curve) ranking for lowest risk of in-hospital mortality was for USAT (SUCRA: 85.15) followed by CDT (SUCRA: 84.56), LDT (SUCRA: 45.41), tPA (SUCRA: 32.41) and lastly anticoagulation (SUCRA: 2.46). Rankogram for mortality risk is given in Fig. 4.

### Major bleeding

**Figure 5:**
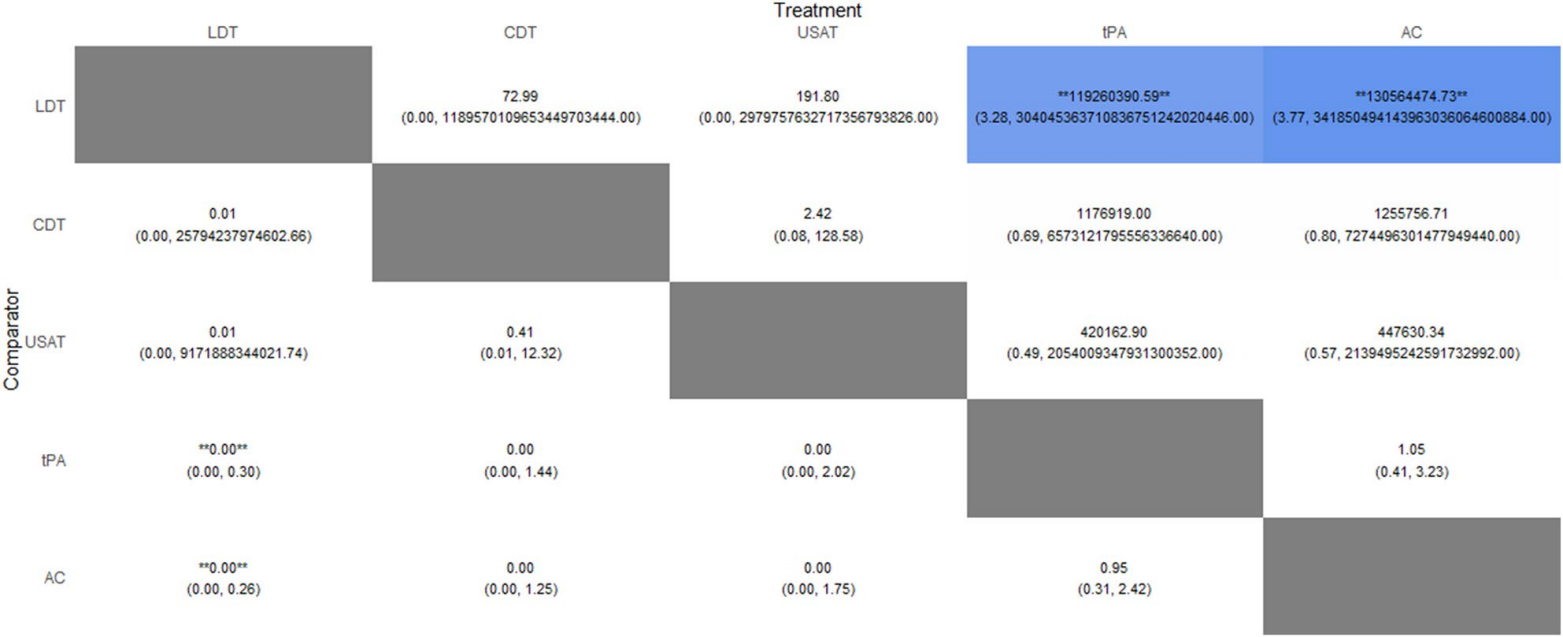
Major Bleeding Heat Plot.

**Figure 6:**
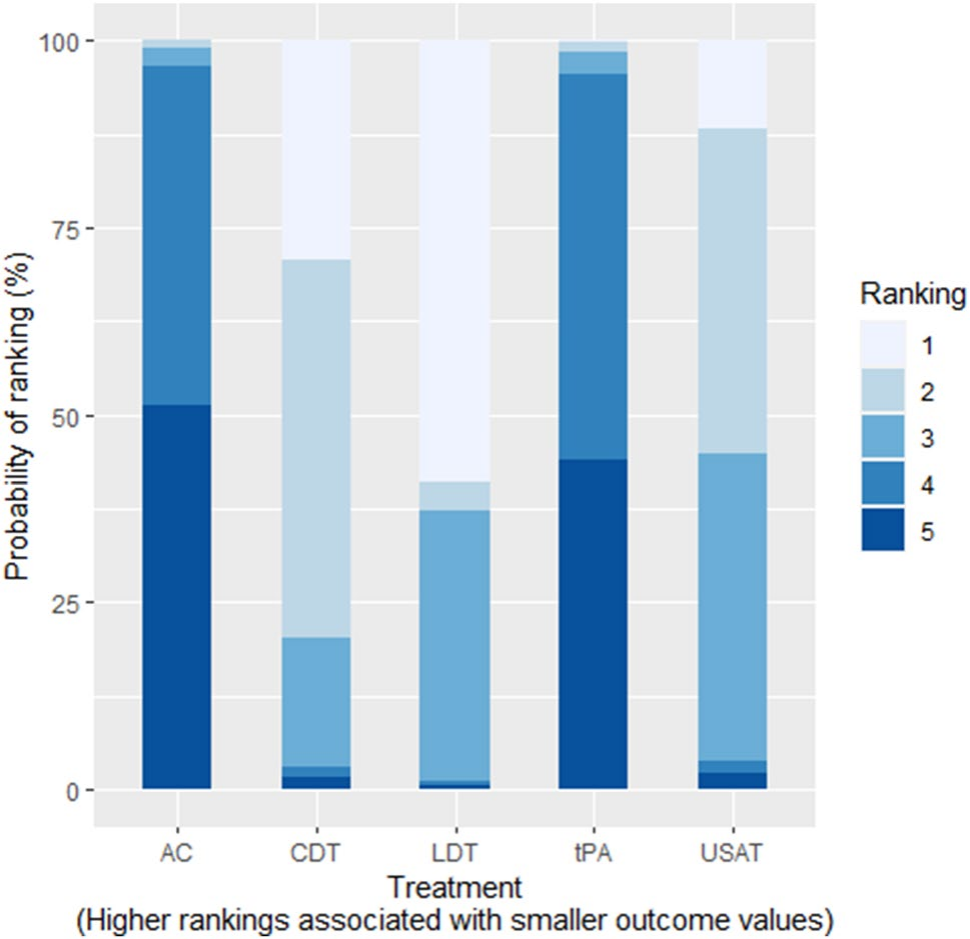
Major Bleeding Rankogram.

Regarding risk of major bleeding, there was no significant difference between tPA and AC (RR: 0.95; 95% CrI: 0.31–2.42) and CDT versus USAT (RR:0.41; 95% CrI: 0.01–12.32). No inferences could be drawn from any other comparisons as their 95% credible intervals included either zero as the lower limit or an exceedingly large number as the upper limit due to having zero events in one of the treatment arms. The Heat Plot is given in Fig. 5. On meta-regression, publication year was not found to have a significant influence (p = 0.36). The SUCRA (surface under the cumulative ranking curve) ranking for lowest risk of major bleeding was for LDT (SUCRA: 80.04), followed by CDT (SUCRA: 76.08), USAT (SUCRA: 65.17), tPA (SUCRA: 15.51) and AC (SUCRA: 13.21). Rankogram is given in Fig. 6.

### Secondary outcomes

#### Minor bleeding

**Figure 7:**
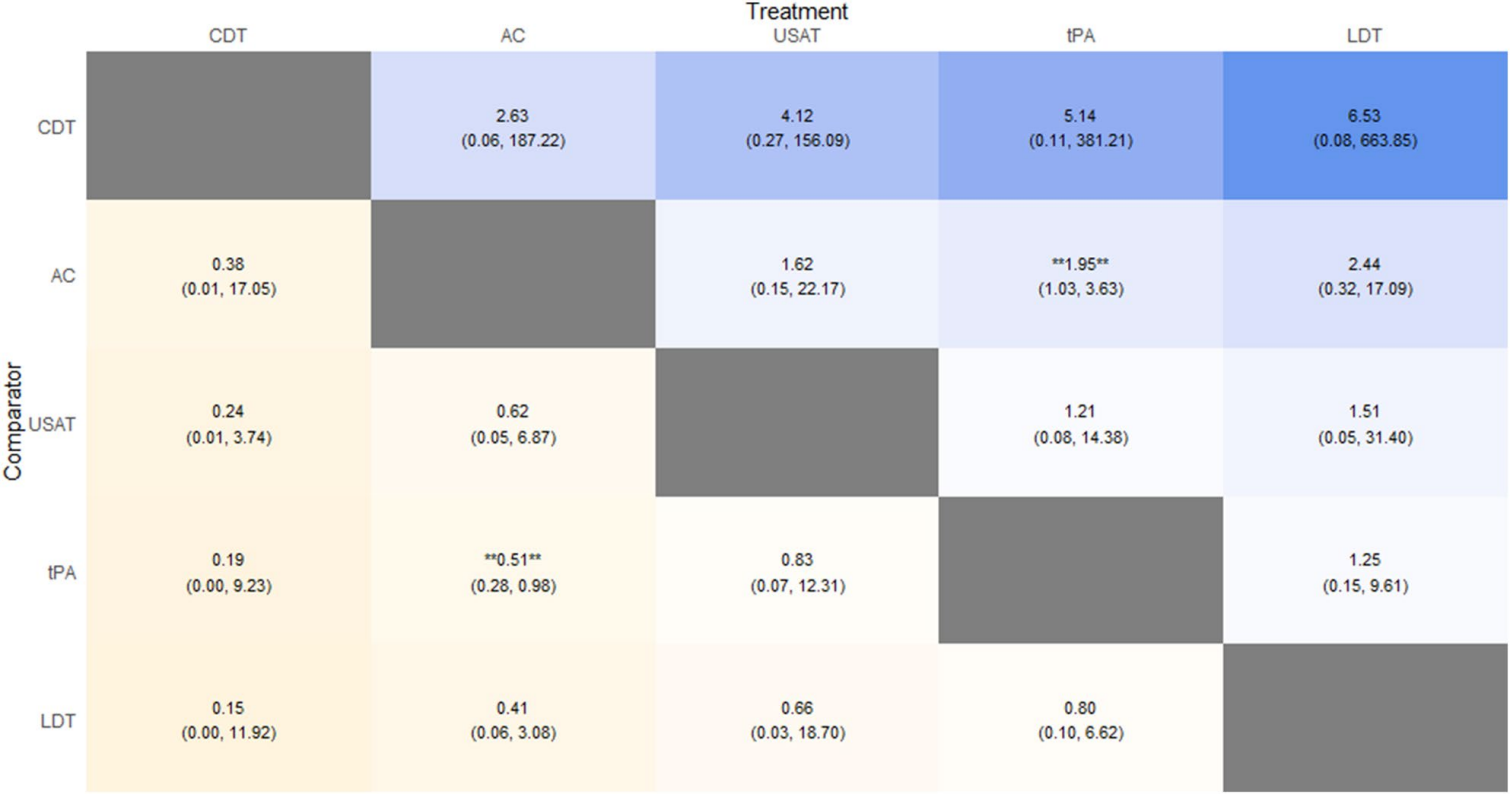
Minor Bleeding Heat Plot.

Fourteen studies comprising 2132 patients were included in estimating the risk of minor bleeding. tPA was found to have significantly higher risk of minor bleeding compared to AC (RR: 1.95; 95% CrI: 1.03–3.63). There were no significant differences between tPA versus USAT, tPA versus LDT, LDT versus CDT, LDT versus AC, LDT versus USAT, USAT versus CDT, USAT versus AC, and CDT versus AC. No inferences could be made between CDT versus LDT and CDT versus tPA. Heat Plot is given in Fig. 7. The SUCRA ranking for lowest risk of minor bleeding was for CDT (SUCRA: 78.73), followed by AC (SUCRA: 69.22), USAT (SUCRA: 41.62), tPA (SUCRA: 31.25) and LDT (SUCRA: 29.18).

#### Recurrent pulmonary embolism

**Figure 8:**
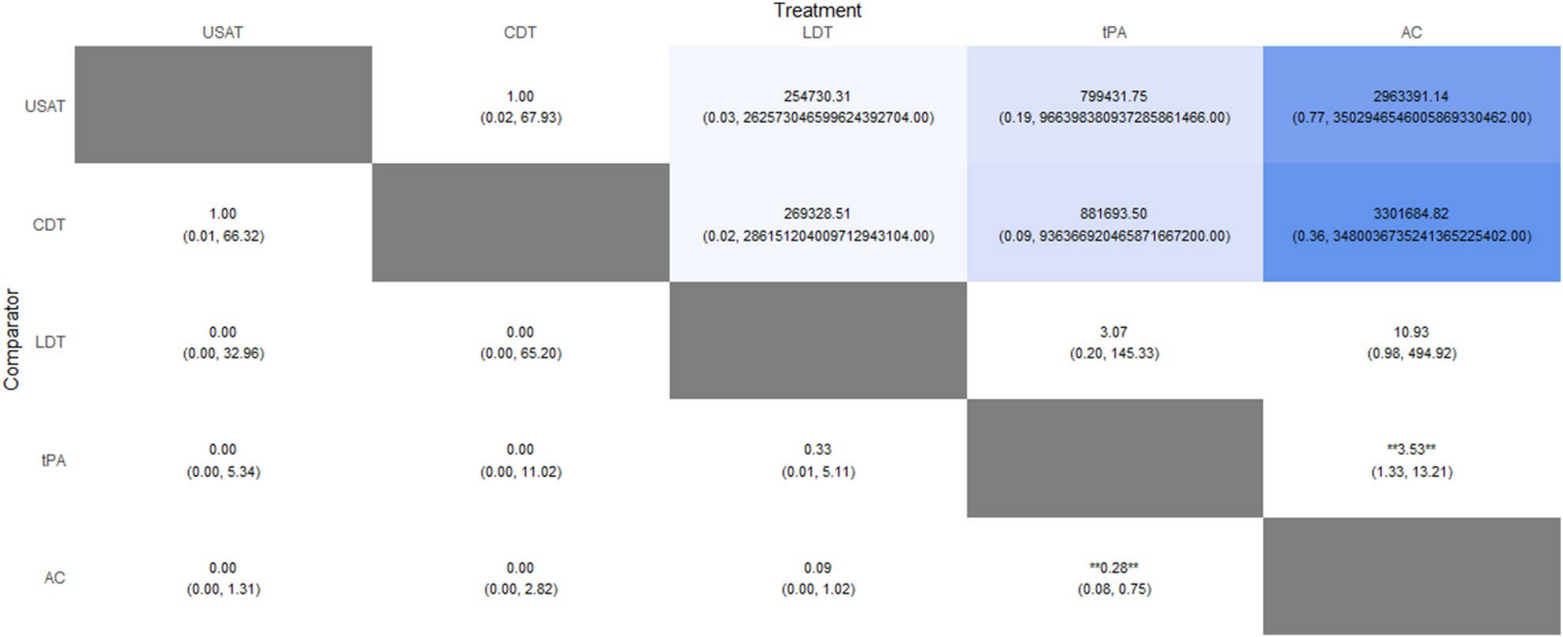
Recurrent PE Heat Plot.

2082 patients from thirteen studies were included. tPA was found to have a lower risk of recurrent PE compared to AC (RR: 0.28; 95% CrI: 0.08–0.75). There was no difference between tPA versus LDT and USAT versus CDT. No inferences could be made from any other comparison. The heat plot is given in Fig. 8. The SUCRA ranking for lowest risk was for USAT (SUCRA: 82.6), followed by CDT (SUCRA: 82.04), LDT (SUCRA: 49.37) and tPA (SUCRA: 33.39). The highest risk for recurrent PE was with AC (SUCRA: 2.59).

#### Risk of bias

Within-study risk of bias was assessed using the Cochrane risk of bias assessment tool for RCTs. All included studies were of good quality and with low risk of bias. We did not find any bias for selective reporting within studies. To assess the risk of bias due to missing evidence we used the ROB-MEN tool^24^. It is a novel tool that incorporates qualitative and quantitative methods, developed by authors involved with Cochrane reviews and methods groups. With ROB-MEN, we initially assess within study bias between pairwise comparisons followed by qualitative assessment of publication bias and quantitative assessment of publication bias. Thereafter, it runs a network meta-regression using the smallest observed variance as a covariate to estimate small study effects and finally calculate the overall risk of bias.

For across study assessment of bias, for qualitative assessment of publication bias, we made the presumption that procedures and newer techniques are more favored. In quantitative assessment of bias, we considered the contribution from evidence to be substantially biased if the difference between treatment groups was at least 15%. On analysis, the overall risk of bias for comparisons were either “low risk” or had “some concerns”. None of the comparisons were found to have high risk of bias. The details of risk of bias assessment is given in the supplemental file.

## Discussion

While anticoagulation has been the main treatment option for patients with submassive (intermediate risk) pulmonary embolism, the PEITHO trial demonstrated that systemic thrombolysis prevented death or hemodynamic deterioration but at the expense of increased risk of bleeding^5^. A meta-analysis that looked at outcomes with thrombolytic therapy versus anticoagulation had shown lower mortality risk with systemic thrombolysis but at the expense of major bleeding risk^25^. Catheter directed thrombolytic methods using either a pigtail catheter or ultrasound assisted thrombolysis were developed to improve mortality rates while decreasing risk of bleeding. This is also the theoretical reasoning behind administering lower dose thrombolytics systemically. The efficacy of catheter directed thrombolytic methods in improving cardiovascular hemodynamics was demonstrated in the ULTIMA trial, SEATTLE II trial and from initial results from PERFECT registry^8,9,26^. But, despite having data on the safety and efficacy of catheter directed methods, randomized controlled trial data on these techniques is scarce. It is also not clear if ultrasound assistance provides greater benefit. The OPTALYSE- PE trial found lower doses of tPA to be effective with USAT, but this was a single arm study^27^. The only randomized controlled trial comparing USAT and CDT to date is the SUNSET sPE trial, which was performed in patients with submassive PE; There was no difference in safety outcomes between the two groups and standard CDT showed better hemodynamic outcomes^11^. The MOPETT trial that compared LDT versus AC found patients in LDT group to have significant reduction in pulmonary artery pressure^17^.

To the best of our knowledge, ours is the first study comparing full dose systemic thrombolysis versus low dose thrombolysis versus standard catheter directed thrombolysis versus ultrasound assisted catheter thrombolysis versus anticoagulation alone in the treatment of acute submassive (intermediate-high risk) pulmonary embolism. In our Bayesian network meta-analysis, significant reduction in mortality was noted in tPA versus AC. There was no significant difference between USAT versus CDT. No inferences could be drawn between any other comparisons. Regarding risk of major bleeding, there was no significant difference in relative risk of major bleeding between tPA versus AC and USAT versus CDT. No inferences could be drawn from any other comparisons. We also found that systemic thrombolysis has a significantly lower risk for recurrent PE compared to anticoagulation but there is a higher risk of minor bleeding complications. Our study differs from the previously published meta-analysis in that we found no significant difference in risk of major bleeding between systemic thrombolysis and anticoagulation.

Our study also shows that while the newer modalities of treatment for pulmonary embolism are promising, there is lack of data to comment on the purported advantages. The available studies are small sized and thereby lack power to demonstrate any statistical significance. There are two ongoing trials that seek to clarify this: HI-PEITHO and PEITHO-3. These studies are estimated to be completed by 2025 and 2027 respectively. The Higher- Risk Pulmonary Embolism Thrombolysis (HI-PEITHO) study is a multinational multicenter randomized controlled trial comparing ultrasound assisted thrombolysis plus anticoagulation (USAT) versus anticoagulation (AC) alone in intermediate-high risk pulmonary embolism^28^. PEITHO- 3 trial is a multinational multicenter randomized controlled trial that compare the efficacy and safety of reduced dose alteplase (LDT) with standard heparin anticoagulation^29^ (AC). The results of these highly anticipated trials might shed light on this important topic.

## Methods

This systematic review and meta-analysis is reported according to the Preferred Reporting Items for Systematic Reviews and Meta-Analysis (PRISMA) Extension statement for systematic reviews incorporating network meta-analyses for health care interventions ^30^.

### Data sources and searches

Literature search was conducted on PubMed/MEDLINE, EMBASE and Cochrane CENTRAL from inception until March 21, 2023. Our librarian (O.S) helped us with devising the search strategy and with literature search. We reviewed grey literature by reviewing scientific research proceedings, conference abstracts and reviewed clinical trials registered on clinical trials.gov. We also screened references of published review articles. The search was not restricted to any language. The search was conducted using keywords and MeSH /Emtree terms:"Pulmonary Embolism"[MeSH Terms] OR "high-risk pulmonary embolism"[All Fields] OR "intermediate-risk pulmonary embolism"[All Fields] OR "acute submassive pulmonary embolism"[All Fields]) AND ("anticoagulant agent"[All Fields] OR "anticoagulants"[MeSH Terms] OR "heparin"[All Fields] OR "heparin"[MeSH Terms] OR "low molecular weight heparin"[All Fields] OR "heparin, low molecular weight"[MeSH Terms] OR "fibrinolytic agent"[All Fields] OR "fibrinolytic agents"[MeSH Terms] OR "thrombolytic therapy"[MeSH Terms] OR "alteplase"[All Fields] OR "urokinase"[All Fields] OR "Urokinase-Type Plasminogen Activator"[MeSH Terms] OR "tenecteplase"[All Fields] OR "tenecteplase"[MeSH Terms] OR "Streptokinase"[All Fields] OR "Streptokinase"[MeSH Terms] OR "tissue plasminogen activator"[All Fields] OR "tissue plasminogen activator"[MeSH Terms] OR "catheter-directed thrombolysis"[All Fields] OR "sonothrombolysis"[All Fields] [Supplemental File].

Search was restricted to adult patients using limit/age filters available on the databases. The articles were downloaded to EndNote and reviewed by two independent reviewers (J.K and B.K), who are both attending physicians in Internal Medicine. Duplicates were identified on EndNote and removed manually. Disagreements between reviewers were resolved with discussion and achieving consensus.

### Study selection

We included randomized controlled trials on adult population that reported at least one relevant clinical outcome of interest for our study. Only studies that specified sub massive (intermediate risk) cases of pulmonary embolism were considered. Sub massive PE was defined as cases with acute PE with objective evidence of RV dysfunction but hemodynamically stable. Evidence of RV dysfunction included positive CT or echo findings and/or elevated cardiac biomarkers such as troponin or BNP or NT-pro BNP. Cases without RV dysfunction were not included in this study. Studies where PE grade was not assessed were not considered. Disagreements between reviewers were resolved by discussion and achieving mutual consensus and after consultation with the lead author (D.M).

### Data extraction

From selected articles, we extracted the number of participants in each treatment arm, total patient sample size, in-hospital mortality, major bleeding, minor bleeding, and recurrent PE events in each group. Major bleeding events were any significant bleeding events as defined by the International Society of Thrombosis and Hemostasis (ISTH)^31^. Bleeding events not fulfilling the definition for major bleeding were counted as minor bleeding events. If clarification were needed regarding the study or variable of interest, corresponding authors were contacted to ensure the study/variables met our case definitions. Quality of studies were assessed using the Cochrane bias risk assessment tool^32^. Risk of bias due to missing evidence was conducted using the ROB-MEN tool^24^.

### Data synthesis and analysis

Data analysis was conducted in R, version 4.1.2, using BUGSnet and R2WinBUGS packages^33^. Outcomes were combined using random effects model using uninformative priors. We specified a burn-in of 50,000 iterations followed by 100,000 iterations with 10,000 adaptations and convergence was checked and confirmed. We estimated the risk ratio (RR) which was reported as 95% credible intervals (CrI). Markov chain Monte Carlo (MCMC) modeling was used to estimate the relative ranking probability of the clinical outcome of interest with each treatment modality and a rankogram was created based on surface under the cumulative ranking curve (SUCRA). Beta-binomial regression was used to handle double-zero studies. Inconsistency was assessed based on the inconsistency model described in the NICE-DSU TSD 4^34^. Here, inconsistency model is weighted against how well they fit the data compared to the consistency model to determine if there is evidence of inconsistency. Correlation plots were created using the posterior mean deviances of each data point between the consistency and inconsistency model (Supplemental File). As we included studies spanning over three decades, we conducted a meta-regression to assess if publication year was an influencing factor.

## Supplementary Information

Supplementary Information.

## Data Availability

All data generated or analyzed during this study are included in this published article and its supplementary information files.
