# Supplementary material for: Mortality and bleeding associated with the management of sub-massive pulmonary embolism: a systematic review and Bayesian network meta-analysis
Source: Sci Rep. 2023 May 3;13:7169. doi: 10.1038/s41598-023-34348-9 (PMC10156731; doi:10.1038/s41598-023-34348-9)

| Pairwise comparison | Number of studies in each comparison (total reported outcomes) | Within-study assessment of bias | Across-study  Assessment of bias | Initial Judgement |
| --- | --- | --- | --- | --- |
| **Observed Comparisons** | | | | |
| AC: LDT | 1 (121) | No bias detected | Suspected bias  favoring LDT | Suspected bias  Favoring LDT |
| AC: tPA | 10 (1805) | No bias detected | Suspected bias favoring tPA | Suspected bias favoring tPA |
| AC: USAT | 1(59) | No bias detected | Suspected bias favoring USAT | Suspected bias favoring USAT |
| CDT: USAT | 1(81) | No bias detected | Suspected bias favoring USAT | Suspected bias favoring USAT |
| **Unobserved Comparisons** | | | | |
| AC: CDT | NA | NA | Suspected bias favoring CDT | Suspected bias favoring CDT |
| CDT: LDT | NA | NA | Suspected bias favoring CDT | Suspected bias favoring CDT |
| CDT: tPA | NA | NA | Suspected bias favoring CDT | Suspected bias favoring CDT |
| LDT: tPA | NA | NA | Suspected bias favoring LDT | Suspected bias favoring LDT |
| LDT: USAT | NA | NA | Suspected bias favoring USAT | Suspected bias favoring USAT |
| tPA: USAT | NA | NA | Suspected bias favoring USAT | Suspected bias favoring USAT |

**Risk of Bias Assessment (ROB MEN)**

**Step 1. Pairwise Comparison Table**

**Step 2. Risk of Bias Assessment in NMA**

| NMA estimate | % Contribution of evidence from pairwise comparisons with suspected bias | | Evaluation of contribution from evidence with suspected bias | Bias assessment for indirect assessment | NMA  Treatment  effect | NMR  Treatment effect at the smallest observed variance | Small study  effects | Overall  Risk of bias |
| --- | --- | --- | --- | --- | --- | --- | --- | --- |
|  | Favoring first treatment | Favoring second treatment |  |  |  |  |  |  |
| **Mixed/only direct** |  |  |  |  |  |  |  |  |
| AC: LDT | 0 | 100 | Substantial contribution favoring LDT | NA | 5.52 (0.26 to 384.70) | 2.32 (0.02 to 536.77) | No evidence of small  Study effects | Some concerns of bias |
| AC: tPA | 0 | 100 | Substantial contribution favoring tPA | NA | 2.85 (1.22 to 11.06) | 1.06 (0.21 to 4.96) | No evidence of small  Study effects | Some concerns of bias |
| AC: USAT | 0 | 100 | Substantial contribution favoring USAT | NA | 131368505.78 (6.15 to 3082064877318614528.00) | 11368629.32 (0.03 to 95501366501681107866484736.00) | No evidence of small  Study effects | Some concerns of bias |
| CDT: USAT | 0 | 100 | Substantial contribution favoring USAT | NA | 0.99 (0.02 to 94.10) | 0.89 (0.00 to 23278.26) | No evidence of small  Study effects | Some concerns of bias |
| **Indirect** |  |  |  |  |  |  |  |  |
| AC: CDT | 0 | 0 | No substantial contribution from bias | Suspected bias favoring CDT | 144364083.56 (2.34 to 3770623907030343680.00) | 9827218.50 (0.05 to 189987808310362817163165696.00) | No evidence of small  Study effects | Low risk of bias |
| CDT: LDT | 0 | 33 | Substantial contribution favoring LDT | Suspected bias favoring CDT | 0.00 (0.00 to 3.46 | 0.00 (0.00 to 49.96) | No evidence of small  Study effects | Some concerns of bias |
| CDT: tPA | 0 | 33 | Substantial contribution favoring tPA | Suspected bias favoring CDT | 0.00 (0.00 to 1.54) | 0.00 (0.00 to 17.86) | No evidence of small  Study effects | Some concerns of bias |
| LDT: tPA | 50 | 50 | No substantial contribution from bias | Suspected bias favoring LDT | 0.52 (0.01 to 16.99) | 0.46 (0.00 to 59.28) | No evidence of small  Study effects | Low risk of bias |
| LDT: USAT | 50 | 50 | No substantial contribution from bias | Suspected bias favoring USAT | 19286122.97 (0.55 to 531249216325893440.00) | 3897562.97 (0.01 to 58450239996356928259227648.00) | No evidence of small  Study effects | Low risk of bias |
| tPA: USAT | 50 | 50 | No substantial contribution from bias | Suspected bias favoring USAT | 49393902.97 (1.84 to 793217851358743168.00) | 9679143.40 (0.03 to 104625013815985007788818432.00) | No evidence of small  Study effects | Low risk of bias |

Search Strategy Used for Literature Search

PubMed/ Cochrane Central

Keywords:

- High risk pulmonary embolism

- Intermediate risk pulmonary embolism

- Acute submassive pulmonary embolism

- MeSH: Pulmonary Embolism

- Anticoagulant agent

o MeSH: anticoagulants

- Heparin

o MeSH: heparin

- Low molecular weight heparin

o MeSH: heparin, low-molecular-weight

- Fibrinolytic agent

o MeSH: fibrinolytic agents

o Thrombolytic therapy

- Alteplase

- Urokinase

- Tenecteplase

- Streptokinase

- Tissue plasminogen activator

o MeSH: tissue plasminogen activator

- Catheter directed thrombolysis

- Sonothrombolysis

("Pulmonary Embolism"[MeSH Terms] OR "high-risk pulmonary embolism"[All Fields] OR "intermediate-risk pulmonary embolism"[All Fields] OR "acute submassive pulmonary embolism"[All Fields]) AND ("anticoagulant agent"[All Fields] OR "anticoagulants"[MeSH Terms] OR "heparin"[All Fields] OR "heparin"[MeSH Terms] OR "low molecular weight heparin"[All Fields] OR "heparin, low molecular weight"[MeSH Terms] OR "fibrinolytic agent"[All Fields] OR "fibrinolytic agents"[MeSH Terms] OR "thrombolytic therapy"[MeSH Terms] OR "alteplase"[All Fields] OR "urokinase"[All Fields] OR "Urokinase-Type Plasminogen Activator"[MeSH Terms] OR "tenecteplase"[All Fields] OR "tenecteplase"[MeSH Terms] OR "Streptokinase"[All Fields] OR "Streptokinase"[MeSH Terms] OR "tissue plasminogen activator"[All Fields] OR "tissue plasminogen activator"[MeSH Terms] OR "catheter-directed thrombolysis"[All Fields] OR "sonothrombolysis"[All Fields])

Embase search

( 'high risk pulmonary embolism'OR 'intermediate risk pulmonary embolism'OR 'acute submassivepulmonary embolism') AND ('anticoagulant agent'/exp OR 'anticoagulant agent'OR 'heparin'/exp OR 'heparin'OR 'low molecular weight heparin'/exp OR 'low molecular weight heparin'OR 'fibrinolyticagent'/exp OR 'fibrinolytic agent'OR 'alteplase'/exp OR 'alteplase'OR 'urokinase'/exp OR 'urokinase'OR 'tenecteplase'/exp OR 'tenecteplase'OR 'streptokinase'/exp OR 'streptokinase'OR 'tissueplasminogen activator'/exp OR 'tissue plasminogen activator'OR 'catheter directedthrombolysis'/exp OR 'catheter directed thrombolysis'OR 'sonothrombolysis'/exp OR'sonothrombolysis) AND ([adult]/lim OR [young adult]/lim OR [middle aged]/lim OR [aged]/lim OR [veryelderly]/lim)

Table. Clinical Outcomes

| **Author year** | **Treatment groups** | **In-hospital Mortality** | **Major Bleeding** | **Minor**  **Bleeding** | **Recurrent PE** |
| --- | --- | --- | --- | --- | --- |
| Levine1990 | tPA vs AC | tPA (1/33)  AC (0/25) | tPA (0/33)  AC (0/25) | tPA (6/33)  AC (1/25) | tPA (0/33)  AC (0/25) |
| Dalla-Volta 1992 | tPA vs AC | tPA (2/20)  AC (1/16) | tPA (3/20)  AC (2/16) | tPA (11/20)  AC (4/16) | tPA (1/20)  AC (3/16) |
| Goldhaber 1993 | tPA vs AC | tPA (0/46)  AC (2/55) | tPA (2/46)  AC (0/55) | tPA (1/46)  AC (1/55) | tPA (0/46)  AC (5/55) |
| Konstantinides 2002 | tPA vs AC | tPA (4/118)  AC (3/138) | tPA (1/118)  AC (5/138) | tPA (72/118)  AC (78/138) | tPA (4/118)  AC (4/138) |
| Becattini 2010 | tPA vs AC | tPA (0/28)  AC (1/30) | tPA (2/28)  AC (1/30) | tPA (13/28)  AC (1/30) | tPA (1/28)  AC (1/30) |
| Fasullo 2011 | tPA vs AC | tPA (0/37)  AC (5/35) | tPA (2/37)  AC (1/35) | tPA (11/37)  AC (4/35) | tPA (0/37)  AC (3/35) |
| Meyer 2014 | tPA vs AC | tPA (6/506)  AC (9/499) | tPA (58/506)  AC (12/499) | tPA (165/506)  AC (43/499) | tPA (1/506)  AC (5/499) |
| Kline 2014 | tPA vs AC | tPA (1/40)  AC (1/43) | tPA (1/40)  AC (0/43) | tPA (1/40)  AC (0/43) | tPA (1/40)  AC (1/43) |
| Taherkhani 2014 | tPA vs AC | tPA (0/25)  AC (3/25) | tPA (0/25)  AC (0/25) | tPA (2/25)  AC (1/25) | N.A |
| Sharifi2013 | LDT vs AC | LDT (1/61)  AC (3/60) | LDT (0/61)  AC (0/60) | LDT (0/61)  AC (0/60) | LDT (0/61)  AC (3/60) |
| Kucher 2014 | USAT vs AC | USAT (0/30)  AC (1/29) | USAT (0/30)  AC (0/29) | USAT (3/30)  AC (1/29) | USAT (0/30)  AC (0/29) |
| Sinha 2017 | tPA vs AC | tPA (2/45)  AC (2/41) | tPA (1/45)  AC ( 1/41) | tPA (7/45)  AC (5/41) | tPA (2/45)  AC (1/41) |
| Zhang 2018 | LDT vs AC | LDT (0/33)  AC (0/33) | LDT (0/33)  AC (0/33) | LDT (8/33)  AC (1/33) | LDT (1/33)  AC (2/33) |
| Avgerinos 2021 | USAT vs CDT | USAT (1/40)  CDT (0/41) | USAT (2/40)  CDT (0/41) | USAT (3/40)  CDT (0/41) | USAT (1/40)  CDT (0/41) |

Supplementary Figures

Supplementary Figure 1. SUCRA Plot of In-hospital Mortality


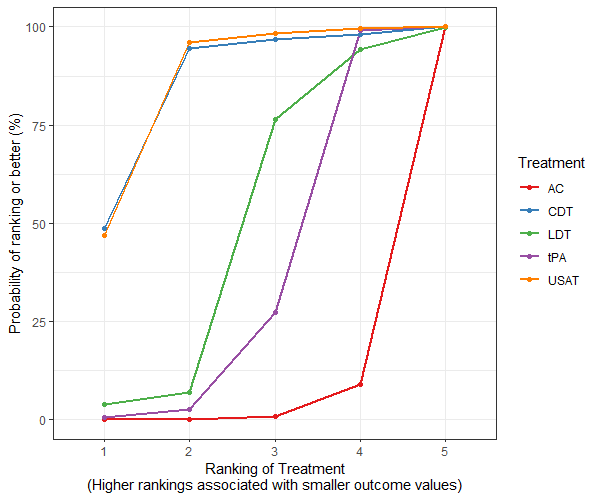


Supplementary Figure 2. SUCRA Plot of Risk of Major Bleeding


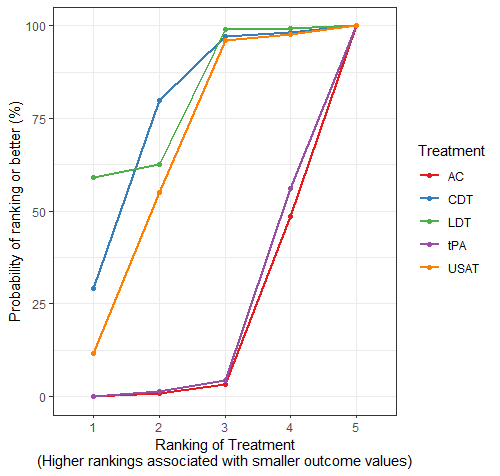


Sensitivity Analysis

A global assessment of transitivity was performed by running an inconsistency model for the NMA as well as a consistency model for the NMA. The posterior mean deviance of each data point between the consistency model and the inconsistency model was then compared on a correlation plot. Leverage plots could not be quantified with imputation of studies with zero events. This was an issue for us as our treatment arms of LDT vs AC , USAT vs AC and USAT vs CDT had one study each, all with zero events in at least one arm. As a result, we failed to estimate DICs. However, this was not an issue on obtaining correlation plots between the consistency model vs the inconsistency model (see below). These plots show a strong linear correlation between the two models suggesting good level of statistical consistency and that transitivity is not violated.

Supplementary Figure 3. Correlation plot (Consistency vs Inconsistency model) on mortality risk


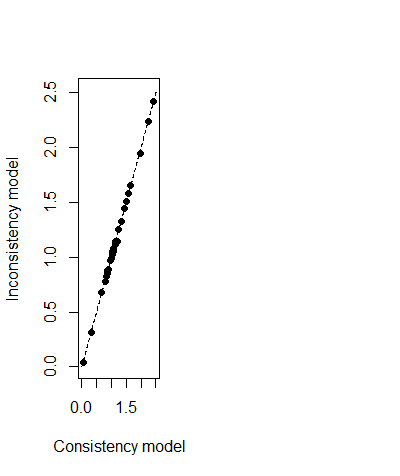


Supplementary Figure 4. Correlation plot (Consistency vs Inconsistency model) on risk of major bleeding


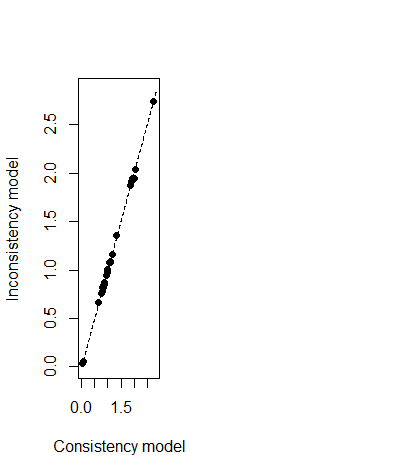


Supplementary Figure 5. Correlation plot (Consistency vs Inconsistency model) on risk of minor bleeding


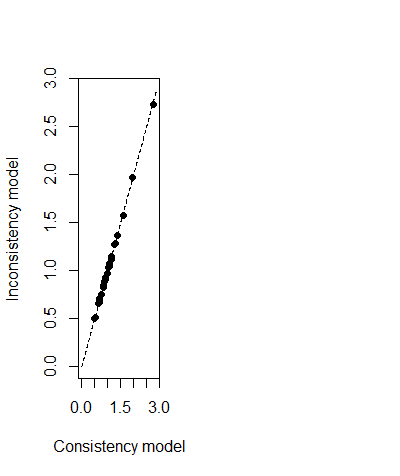


Supplementary Figure 6. Correlation plot (Consistency vs Inconsistency model) on risk of recurrent PE


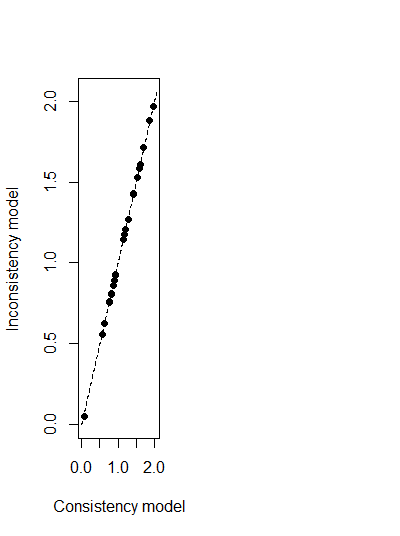

Supplement: Supplementary file 1 — Supplementary Information. [file 41598_2023_34348_MOESM1_ESM.docx]
